# Depression as a Prodromal Symptom of Pancreatic Cancer: A Narrative Review

**DOI:** 10.3390/cancers18010016

**Published:** 2025-12-19

**Authors:** Chiara Deori, Federica Andreis, Valentina Giubileo, Silvia Noventa, Ester Oneda, Fausto Meriggi, Alberto Zaniboni

**Affiliations:** Oncology Department, Istituto Ospedaliero Fondazione Poliambulanza, Via Bissolati 57, 25124 Brescia, Italy; federica.andreis@poliambulanza.it (F.A.); valentina.giubileo@poliambulanza.it (V.G.); silvia.noventa@poliambulanza.it (S.N.); ester.oneda@poliambulanza.it (E.O.); fausto.meriggi@poliambulanza.it (F.M.); alberto.zaniboni@poliambulanza.it (A.Z.)

**Keywords:** pancreatic cancer, depression, prodromal symptoms, cytokines, kynurenine pathway

## Abstract

Background: Pancreatic ductal adenocarcinoma (PDAC) is one of the most lethal malignancies, typically diagnosed at an advanced stage. The identification of prodromal symptoms could support earlier detection. Among these, depression is frequently reported, raising the question of whether it may represent not only a reactive response but also a paraneoplastic manifestation. Methods: We conducted a narrative review of clinical, epidemiological and biological literature published between 1988 and 2025. Searches were performed in PubMed/MEDLINE, Scopus, and Web of Science using predefined keywords related to pancreatic cancer, depression, prodromal symptoms, cytokines, and the kynurenine pathway. Eligible studies included clinical cohorts, population-based analyses, biological investigations, and case reports exploring the temporal or mechanistic link between depression and PDAC. Results: A substantial proportion of patients (10–20%) exhibit depressive symptoms in the months preceding the clinical diagnosis of pancreatic cancer. In several cases, depression occurs independent of weight loss and new-onset diabetes. Biological evidence highlights the involvement of pro-inflammatory cytokines (IL-6, IL-1β, TNF-α), NF-κB signaling, and activation of the tryptophan–kynurenine pathway (IDO), suggesting a link between tumor-related processes and mood alterations. These mechanistic findings are actually hypothesis-generating, deriving mainly from small clinical cohorts and preclinical models. Clinically, depression is associated with reduced adherence to treatment, poorer quality of life, and shorter survival. However, no specific depressive phenotype has been identified. Conclusions: Depression may represent a potential prodromal symptom of pancreatic cancer, reflecting systemic biological processes as well as psychological reactions. Its utility as a standalone marker remains limited; future studies should integrate psychiatric, clinical and biological biomarker assessments to enhance early clinical diagnosis.

## Simple Summary

Depression is frequently observed in patients with pancreatic ductal adenocarcinoma (PDAC) and may appear even months before the cancer is diagnosed. This narrative review summarizes clinical, epidemiological and biological evidence suggesting that depression could represent a prodromal or paraneoplastic manifestation of pancreatic cancer, rather than only an emotional reaction to illness. Understanding this association may help clinicians recognize early warning signs and improve patient care, prognosis, and quality of life. This review primarily focused on PDAC; evidence in other pancreatic tumor histotypes is limited to isolated reports.

## 1. Introduction

Pancreatic cancer (PC) ranks among the most aggressive and lethal malignancies: the 5-year survival rate remains below 12%, and diagnosis is often established at an advanced stage [1,2]. Over the past decades, both incidence and mortality have more than doubled worldwide; the main risk factors include cigarette smoking, obesity, and hyperglycemia [2]. In Italy, AIOM 2024 data confirm a steady increase in incidence of PC from 2014 to 2023, highlighting the substantial epidemiological burden [1]. The lack of effective screening tools makes the identification of early warning signs, or prodromal symptoms, crucial to facilitate timely diagnosis. In recent years, alongside established conditions such as weight loss and new-onset diabetes [3], increasing attention has been directed toward depression as a potential paraneoplastic manifestation of PC.

The prevalence of depression among PC patients ranges from 33% to 71%, and exceeding that observed in other gastrointestinal malignancies [4,5,6]. As early as the 1930s—and subsequently in the pioneering works of Shakin & Holland and Makrilia and colleagues—depression was reported as a frequent and sometimes early symptom [7,8]. Mayr & Schmid described a distinctive feature in some patients: the peculiar “sense of impending doom” [9]. More recent narrative reviews [5,6] have underscored that depression should not be regarded solely as a psychological reaction to diagnosis, but rather as a potential reflection of an intrinsic biological mechanism.

Epidemiological evidence reinforces this hypothesis: population-based studies [10,11] have documented that, in a substantial proportion of cases, depressive symptoms precede clinical diagnosis. Similarly, Olson and colleagues, in a case–control study of over 500 patients, demonstrated that depression and fatigue were significantly more frequent among cases than controls, independently of other paraneoplastic conditions [3]. Moreover, recent analyses [12,13] indicate that prodromal depression is associated with poorer treatment adherence and worse prognosis. Boyd, in earlier work, and more recently Chen and collaborators, confirmed that depression—whether preexisting or concomitant with diagnosis—correlates with more advanced disease stages and reduced survival [4,14].

In parallel, numerous studies investigating biological markers suggest a shared pathophysiological substrate between tumor progression and mood alterations. Torres [15], Breitbart [16], and Jarrin Jara [17] documented elevated levels of IL-6, IL-1β, and TNF-α in PC patients, associated with depressive symptoms, albeit in preliminary findings. Activation of the NF-κB pathway has been identified as a key driver of inflammation and pancreatic carcinogenesis [18]. Furthermore, research on the tryptophan–kynurenine pathway, mediated by the enzyme IDO1, has revealed serotonin depletion and the accumulation of neurotoxic metabolites as possible mediators of tumor-associated depression [19,20,21]. More recently, studies have also proposed the involvement of the gut–brain axis and intestinal dysbiosis [22].

From a psychosocial perspective, it is well established that depression in PC patients is linked to poorer quality of life [23], higher caregiver distress [24], and even an increased risk of suicide, particularly in the first months after diagnosis [25].

In summary, an extensive and growing body of clinical literature suggests that depression should not be regarded merely as a psychological consequence of PC diagnosis, but rather as a potential prodromal symptom and a biologically mediated paraneoplastic manifestation.

PDAC differs from many other solid tumors not only because of its aggressive biological behavior, but also because of the paucity of specific early symptoms and the rapid clinical deterioration that often precedes diagnosis. Unlike malignancies in which psychological distress typically emerges as a reaction to diagnosis or treatment, PC has long been characterized by the unusual frequency of affective symptoms occurring before the clinical recognition of the disease [6,9]. This distinctive temporal pattern raises important questions regarding the nature of depression in this context and challenges the traditional distinction between reactive and disease-related mood disorders.

Depression as a potential prodromal manifestation of PC represents a particularly complex clinical phenomenon, as it overlaps with common psychiatric conditions in the general population and lacks specific diagnostic features. Nevertheless, its recurrent association with PC across epidemiological, clinical, and biological studies suggests that it may reflect underlying tumor-related processes rather than a coincidental comorbidity [6].

Clarifying the role of depression in the early phases of PC is clinically relevant for several reasons. First, it may contribute to earlier recognition of high-risk patients when interpreted in conjunction with other warning signs. Second, untreated depressive symptoms may negatively influence help-seeking behavior, diagnostic pathways, and therapeutic decision-making. Finally, understanding whether depression represents a paraneoplastic phenomenon may open new perspectives for integrated oncological and psychiatric care [9].

This narrative review aims to integrate these findings, critically evaluating the role of depression as an early marker of PC and its clinical, prognostic, and translational implications.

## 2. Materials and Methods

A narrative review of the literature published between 1988 and 2025 was conducted following recognized methodological guidelines for narrative syntheses. Searches were performed in PubMed/MEDLINE, Scopus, and Web of Science, using the Boolean string: (“pancreatic cancer” OR “pancreatic neoplasm”) AND (“depression” OR “mood disorders”) AND (“prodromal symptoms” OR “paraneoplastic”) AND (“cytokines” OR “IL-6” OR “TNF-α” OR “inflammation” OR “kynurenine” OR “IDO1”).

We included clinical, epidemiological, and biological studies, as well as case reports and reviews in English or Italian that explored the relationship between PC and depression from psychological, biological, or temporal perspectives. Non-peer-reviewed abstracts, purely theoretical papers, and non-human studies without translational relevance were excluded.

**Figure 1:**
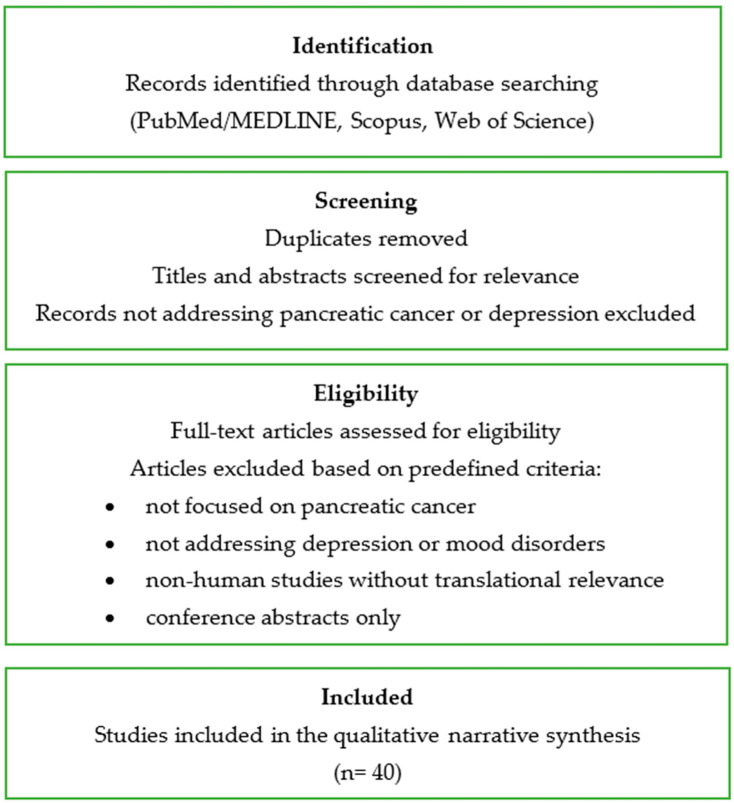
Flow diagram of the study selection process.

The study selection process is summarized in a flow diagram to enhance transparency of the narrative review methodology (Figure 1).

Titles, abstracts and full texts were screened, and reference lists of included papers were examined to identify additional relevant articles. A descriptive synthesis of evidence across epidemiological, clinical, and experimental domains was then performed.

## 3. Results

### 3.1. Epidemiological Aspects

PC is the seventh leading cause of death worldwide and one of the fastest-growing malignancies in terms of incidence and mortality [20,26,27]. Multiple lines of evidence support this: in Italy, according to the 2024 AIOM report [1], approximately 14,500 new diagnoses are expected, with a steadily increasing trend. Data from the Global Burden of Disease Study confirm a doubling of cases and deaths between 1990 and 2017, underscoring the importance of modifiable risk factors such as smoking, elevated blood glucose, and obesity [2].

From a psychological standpoint, the prevalence of depressive symptoms in PC patients is estimated between 33% (diagnosed major depression) and 71% (depressive symptoms), a significantly higher proportion compared to that observed in other gastrointestinal cancers [5,6]. Historically, a distinctive association between PC and mood disorders had already been noted [7,8]), later expanded by studies suggesting that depression may not simply be a psychological reaction to diagnosis but could also exhibit paraneoplastic characteristics, as hypothesized in early conceptual reviews [5,9].

Several population-based epidemiological studies have examined the timing of depressive symptom onset (Table 1). In a large national study, Seoud et al. [11] reported that 16.4% of PC patients had a diagnosis of depression prior to their cancer diagnosis, compared to 13% afterward, suggesting that depression may precede the clinical manifestation of the disease, though not to a statistically significant degree. Ferreira et al. [10] reported in clinical cases analyses that depressive episodes occurring before the emergence of physical symptoms, contributing to diagnostic delays.

Across studies, the pre-diagnostic temporal window for depressive symptoms varies, but it is most consistently reported within the 6 months preceding PC diagnosis; some reports describe onset up to 12 months earlier. Importantly, the extent to which studies distinguished new-onset depression during suspected tumor growth from pre-existing mood disorders differed across datasets: administrative cohorts typically relied on diagnostic codes to identify prior affective or bipolar disorders, whereas several clinical cohorts did not systematically assess psychiatric history. This heterogeneity limits the certainty with which prodromal depression can be separated from pre-existing affective illness.

Olson and colleagues [3], in a case–control study conducted at Memorial Sloan Kettering Cancer Center, demonstrated that fatigue and depression were significantly more common among PC patients than controls, independently of other paraneoplastic conditions such as new-onset diabetes and weight loss. Similarly, Davis and his group [12] showed that the presence of depression and anxiety in the prodromal phase was associated (not causally) with lower treatment adherence and reduced survival.

Finally, Boyd et al. [4] and Ji et al. [13] reported that depression documented prior to PC diagnosis is associated with more advanced disease at presentation, lower likelihood of receiving appropriate treatments, and poorer survival outcomes. However, the distinction between pre-existing and new-onset depression was not uniform across studies, reinforcing the need for prospective cohorts with standardized psychiatric assessment.

### 3.2. Depression as a Prodromal Symptom of Pancreatic Cancer

In addition to epidemiological data, numerous clinical findings reinforce the hypothesis that depression may represent a prodromal symptom of PC. Traditionally, weight loss, new-onset diabetes, and asthenia have been recognized as possible early warning signs of PC [3,28]. Olson et al. [3], in a case–control study, demonstrated that depression and fatigue were significantly more common in PC patients than in controls, independently of weight loss and diabetes, suggesting an autonomous role of depression as a paraneoplastic manifestation. Kenner [28] emphasized that anxiety and depression may precede diagnosis and could serve as potential clinical “red flags.”

Clinical studies and case reports confirm that depression may constitute the first manifestation of PC. Ferreira and colleagues [10] described a patient with severe depression preceding physical symptoms, leading to a delayed diagnosis. Similarly, Barnes et al. [29] reported clinical cases in which depression was the presenting symptom of PC. Villa et al. [30], in a single case report of pancreatic neuroendocrine tumor (PNET), observed the onset of depression prior to the oncological diagnosis, supporting the prodromal role even in less frequent histotypes. Several authors have described distinctive features of depression in PC. Mayr and Schmid [9] reported the qualitative observation of a “sense of impending doom” as a specific clinical element, experienced by some patients in the early phase. Shakin and Holland [8], in one of the earliest systematic analyses, had already reported the high prevalence of depressive disorders among PC patients and the possibility that they preceded cancer diagnosis.

### 3.3. Clinical Course and Prognostic Implications

The trajectory of depressive symptoms in PC has also been evaluated in relation to treatment. Sato et al. [31] found that depression tends to persist from the preoperative phase up to six months after surgical resection, often associated with pain and gastrointestinal symptoms. Ibrahim et al. [23], in a small prospective cohort, documented that newly diagnosed patients already reported high levels of psychological distress, fatigue, and depressive symptoms at presentation, with significant impact on quality of life. Depression is not only a potential prodromal symptom but also influences subsequent clinical outcomes. Boyd et al. [4] showed that preexisting depression is associated with diagnosis at more advanced stages and a lower likelihood of receiving appropriate treatments, while Ji et al. [13] confirmed the association with reduced survival and poorer quality of life. Davis et al. [12] further observed that pre-diagnosis depression and anxiety are associated with lower treatment adherence, negatively affecting outcomes.

### 3.4. Hypothesized Biological Mechanisms Linking Depression and Pancreatic Cancer

Understanding the link between depression and PC faces a substantial limitation: the lack of reliable biomarkers capable of distinguishing prodromal depression from reactive depression at an early stage [5]. To date, no validated biomarker exists for clinical application. The prevailing hypothesis is that affective symptoms may represent paraneoplastic manifestations—expressions of hypothesized measurable biological processes—rather than mere psychological reactions [7,8]). Overall, the mechanistic literature in PC remains preliminary and should be interpreted as hypothesis-generating rather than confirmatory [Table 2].

### 3.5. Systemic Inflammation and Cytokines

The tumor microenvironment in PC is characterized by chronic inflammation with overproduction of cytokines such as IL-6, TNF-α, and IL-1β [5,15,17]. Activation of the IL-6/gp130/JAK-STAT3 pathway, often in synergy with NF-κB, promotes proliferation, angiogenesis, and treatment resistance, while also stimulating the acute-phase response with increased CRP—an unfavorable prognostic, non-diagnostic marker [14,18]. Serological evidence has demonstrated elevated IL-6 levels not only in blood but also in cerebrospinal fluid, suggesting central involvement and a potential—though preliminary—role as a sentinel signal [28]. Consistent with these data, Breitbart et al. [16] observed higher IL-6 concentrations in depressed compared to non-depressed patients, supporting the hypothesis of a possible “cytokine-related” depressive phenotype [32,33].

### 3.6. Immune-Mediated and Neuroendocrine Mechanisms

Beyond the inflammatory model, some authors have proposed specific immune-mediated mechanisms: the formation of antibodies against tumor antigens, with cross-reactivity toward central serotonergic receptors, could reduce serotonin availability and result in depressive syndromes that are resistant to treatment, even if it remains speculative [5,7,8]). This perspective complements, rather than replaces, the cytokine hypothesis.

### 3.7. Tryptophan-Kynurenine Pathway

Another well-established finding is the diversion of tryptophan metabolism toward the kynurenine (KYN) pathway, induced by IFN-γ, TNF-α, and glucocorticoids through the activation of IDO1/IDO2 and TDO [21]. This leads to reduced serotonin synthesis and accumulation of neuroactive metabolites such as quinolinic acid and 3-hydroxykynurenine, with neurotoxic and excitotoxic effects in limbic regions [29]. Both clinical and preclinical studies support this mechanism: interferon-α administration induces IDO1 and depressive symptoms [34]; in orthotopic PC mouse models, IDO1 inhibition reduces KYN levels and depressive-like behaviors, whereas an SSRI produces no improvement [35]. Clinically, higher tumor burden is associated with elevated KYN levels and worse mood scores, with suggestive predictive value of the KYN/Trp and KYNA/Trp ratios [19].

### 3.8. Hormonal and Neuroendocrine Alterations

Endocrine abnormalities have been observed in PC patients, including increased peripheral 5-HTP and serotonin, elevated urinary metabolite 5-HIAA, and multiple endocrine dysfunctions (thyroid, ACTH, ADH) reported in small series [5,7]. These alterations suggest central serotonin depletion, contributing to depressive symptoms. In some cases, depressive disorders resolved only after tumor resection, supporting an organic origin [5].

### 3.9. Paraneoplastic Hypothesis

According to the paraneoplastic model, pancreatic tumors may produce yet-unidentified substances with neurotransmitter-like activity capable of altering mood tone and interfering with the HPA axis [6,7]. Olson et al. [3] proposed that depression and fatigue may constitute a cytokine-mediated paraneoplastic syndrome, independent of diabetes or weight loss.

### 3.10. Chronic Stress and β-Adrenergic Signaling

Preclinical evidence suggests that chronic stress-related mechanisms associated with depressive states may influence PC progression through persistent activation of the sympathetic nervous system: excess catecholamines stimulate β-adrenergic receptors on tumor cells, promoting growth and metastasis [32].

However, clinical evidence supporting a causal role of depressive disorders in PC development or progression remains limited and highly controversial.

### 3.11. Microbiota and the Gut–Brain Axis

Recent studies suggest that intestinal dysbiosis may act as a mediator of cancer-related depressive symptoms. Liu et al. [22] demonstrated, in a radiation enteropathy model, that microbiota disruption increases cytokines, intestinal permeability, and HPA axis activation, thereby promoting depression. Although not specific to PC, these findings are consistent with the shared inflammatory–immune model.

**Table 2:** Key biological pathways between depression and PC.

Author (Year)	Model/Study Type	Marker/ Pathway Analyzed	Main Findings	Implications
[15] Torres et al. (2014)	Serum of PC patients	Cytokine profile	↑ IL-6, TNF-α, IL-1β	Systemic inflammation is linked to depressive symptoms.
[16] Breitbart et al. (2014)	Clinical study	IL-6	Higher levels in depressed vs. non-depressed patients	Supports a “cytokine-related” depressive phenotype.
[17] Jarrin Jara et al. (2020)	Review/clinical analysis	IL-6 and inflammatory cytokines	Direct involvement in PC-associated depression	Confirms paraneoplastic hypothesis.
[18] Silke & O’Reilly (2021)	Biological review	NF-κB	Driver of inflammation and carcinogenesis	Potential therapeutic target.
[14] Chen et al. (2025)	Prospective cohort	Log-transformed CRP	Depression + elevated CRP independently predict reduced OS	Incorporating inflammatory markers into risk stratification.
[21] Sforzini et al. (2019)	Review on the KYN pathway	Tryptophan–kynurenine pathway	Metabolic shift → ↓ serotonin, ↑ neurotoxic metabolites	Depression as an immuno-metabolic disorder.
[19] Botwinick et al. (2014)	Clinical study in PC patients	KYN/Trp ratio	↑ KYN correlated with worse mood	Clinical predictive value.
[15] Torres et al. (2014)	Serum of PC patients	Cytokine profile	↑ IL-6, TNF-α, IL-1β	Systemic inflammation is linked to depressive symptoms.
[16] Breitbart et al. (2014)	Clinical study	IL-6	Higher levels in depressed vs. non-depressed patients	Supports a “cytokine-related” depressive phenotype.
[34] Wichers & Maes (2002)	Clinical study (interferon-α)	IDO1/KYN pathway	IFN-α induces depressive symptoms via IDO1	Translational experimental model.
[35] Hue et al. (2022)	Orthotopic murine models	IDO1 inhibition	↓ KYN + ↓ depressive-like behaviors	Innovative pharmacological target.
[7] Makrilia et al. (2009)	Clinical review	Neuroendocrine alterations	HPA dysfunction, ↑ 5-HIAA, peripheral serotonin	Paraneoplastic contribution.
[22] Liu et al. (2024)	Murine model (radiation enteropathy)	Gut dysbiosis/gut–brain axis	Microbiota alteration → ↑ cytokines, intestinal permeability, HPA activation	Possible role of microbiota in cancer-related depression.

Key biological mechanisms linking depression and PC: inflammatory, immuno-metabolic, neuroendocrine, and gut dysbiosis processes. The arrows indicate an increase or decrease in the reported dimensions.

## 4. Discussion

The association between depression and PDAC has emerged as an area of growing clinical and scientific interest, not only because of the high prevalence of depressive disorders in this population but also due to their prognostic and quality-of-life implications. Increasing evidence suggests that depression should not be regarded as a purely reactive epiphenomenon to cancer diagnosis, but rather may represent a prodromal and, in part, biologically mediated manifestation of the disease.

From a prognostic standpoint, several studies have documented that preexisting or concomitant depression is associated with poorer outcomes, including more advanced clinical presentations at diagnosis, lower likelihood of undergoing surgical or chemotherapeutic treatment, and reduced overall survival [4,13]. These associations appear to reflect multiple mechanisms: on the one hand, biological factors such as inflammatory activation and neuroendocrine dysregulation; on the other, clinical and behavioral variables, including reduced adherence to oncological therapies [12,14]. Preexisting psychiatric disorders have also been associated with higher overall and cancer-specific mortality, as demonstrated by Paredes et al. [36], reinforcing the need for early psychiatric screening in patients at risk of PDAC. The interplay between depressive symptoms and inflammatory markers, such as C-reactive protein, further supports the notion that depression in this context represents a distinct clinical phenotype, suggesting independent prognostic significance.

The impact extends beyond survival, deeply affecting quality of life. PDAC patients with depressive symptoms report significant physical, emotional, and cognitive impairments, often accompanied by anhedonia, insomnia, poor appetite, and concentration difficulties, occasionally progressing to suicidal ideation [23]. This symptomatic burden further exacerbates the illness experience and extends to caregivers, creating an emotional interdependence that underscores the importance of incorporating psychosocial support into treatment pathways [24]. Additional evidence indicates that depression in PDAC patients contributes to a broader picture of psychosocial distress, requiring an integrated clinical approach that also considers preexisting mental health conditions [37].

The observation that depression may precede PDAC diagnosis opens important perspectives for early screening. Recent studies have shown that new-onset depression in adulthood, particularly when associated with symptoms such as weight loss, fatigue, or recent-onset diabetes, may serve as a clinical red flag [3,38]. Although affective symptoms alone cannot be considered specific or sufficient as a stand-alone screening tool, their integration with other clinical and biological indicators could improve risk stratification in selected patient subgroups [28].

In terms of management, depression associated with PDAC is often reported as relatively resistant to conventional antidepressant treatments [6]. Early integrated palliative interventions have demonstrated benefits in terms of pain and quality of life, but not in significantly improving depressive symptoms [39], suggesting that management requires multidimensional approaches combining psycho-oncology, pharmacotherapy, and supportive care. Looking ahead, the integration of inflammatory and immuno-metabolic biomarkers—such as interleukin-6, CRP, and the KYN/Trp ratio—may, in future, help identify more vulnerable patients of more vulnerable patients and open avenues for targeted therapeutic interventions. Preclinical evidence on the tryptophan–kynurenine pathway [35] further supports the hypothesis of innovative pharmacological targets, such as IDO1 inhibition.

However, biological mechanisms alone are insufficient to fully capture the complexity of depressive symptoms observed in PC.

While increasing biological evidence supports a paraneoplastic contribution to depressive symptoms in PDAC, depression in this context cannot be fully understood through a purely biological lens. Rather, it should be conceptualized within a biopsychosocial framework, in which tumor-related biological processes interact with psychological vulnerability, social context, and cultural representations of the disease [6,32].

From a psychological perspective, PDAC is often perceived as a disease characterized by rapid progression, limited therapeutic options, and poor prognosis. Such representations may generate profound existential distress, anticipatory anxiety, and feelings of helplessness even before the communication of a formal clinical diagnosis. Illness representations and perceived loss of control may therefore amplify depressive responses independently of biological mechanisms.

Social and relational dimensions further contribute to psychological burden. Functional decline, early dependency and disruption of social roles may already emerge during the prodromal phase, while caregivers’ distress and unmet supportive needs have been shown to closely mirror patients’ depressive symptoms [24]. Cultural narratives surrounding PC —frequently associated with fatalism and stigma—may also shape emotional responses, coping strategies, and help-seeking behaviors.

Importantly, these psychosocial factors do not oppose biological hypotheses but rather complement them, suggesting that depressive symptoms in PDAC arise from the convergence of systemic inflammation, neuroendocrine alterations, immuno-metabolic pathways, and psychosocial stressors. Recognizing this multidimensional interplay is essential to avoid reductionist interpretations and to inform integrated clinical approaches combining oncological treatment, psycho-oncological care, and early psychiatric assessment.

The recognition of depression as a potential prodromal or paraneoplastic manifestation of PDAC carries important clinical and psycho-oncological implications. From a diagnostic perspective, the identification of new-onset depressive symptoms in adulthood—particularly when accompanied by nonspecific somatic complaints such as fatigue, weight loss, or metabolic alterations—should prompt careful clinical evaluation and longitudinal monitoring. While depression alone cannot justify oncological screening, its presence may contribute to a broader risk stratification framework when integrated with other clinical warning signs and risk indicators [3,28].

From a therapeutic standpoint, early detection and management of depressive symptoms are essential to optimize oncological care. Depression has been consistently associated with reduced treatment adherence, impaired decision-making capacity, and poorer engagement with healthcare services. These factors may partially explain the association between depression and worse survival outcomes observed in PC cohorts [4,12,13]. Systematic psychiatric assessment at the time of clinical suspicion or diagnosis may therefore represent a crucial step in comprehensive patient management.

Psycho-oncological interventions should be integrated early in the disease trajectory, rather than being limited to advanced or palliative stages. Multidisciplinary care models that combine oncological treatment with psychological support, psychiatric evaluation, and symptom management may help mitigate the negative impact of depression on quality of life and functional status. In this context, collaboration between oncologists, psychiatrists, psychologists, and palliative care specialists becomes particularly relevant [23,24].

Importantly, the frequent resistance of PC-associated depression to conventional antidepressant treatments suggests that standard pharmacological approaches may be insufficient when used in isolation. This observation further supports the need for integrated strategies that address both biological and psychosocial contributors to depressive symptoms. Interventions targeting inflammation, pain, fatigue, and existential distress may therefore play a complementary role alongside psychopharmacological and psychotherapeutic approaches [6,35].

Overall, acknowledging depression as an integral component of the PC clinical phenotype may help shift clinical practice toward a more integrated model of care, in which psychological symptoms are not considered secondary outcomes but central elements influencing prognosis, treatment trajectories, and patient-centered outcomes.

Despite these advances, the literature still presents substantial gaps. The prevalence and specificity of prodromal depression in PDAC remain debated, as does the distinction between reactive and paraneoplastic forms, complicated by the lack of validated clinical or biological markers. Similarly, proposed biological mechanisms require confirmation in large prospective cohorts, while therapeutic options specifically addressing depression in PDAC remain limited and only partially effective [23,39]. The growing interest in this field is reflected in the launch of systematic review protocols aimed at clarifying the link between depression, PDAC progression, and clinical outcomes [40].

In summary, depression in PDAC represents both a clinical challenge and a potential opportunity. On one hand, it negatively affects quality of life, treatment adherence, and survival; on the other, it may serve as a potential early indicator of disease and as a target for new integrated therapeutic strategies. Future perspectives call for risk models that combine affective symptoms, biological biomarkers, and artificial intelligence tools to enable earlier clinical diagnosis, along with the development of multidisciplinary protocols integrating oncology, psychiatry, psycho-oncology, and translational research.

### Limitations

This review has some limitations that should be considered when interpreting the findings. As a narrative synthesis, it does not aim to provide a quantitative assessment or a systematic evaluation of study quality. The available evidence on prodromal depression in PC remains heterogeneous in terms of study design, timing of symptom assessment, and operational definitions of depression, which may partly explain variability across results; assessment of depression was heterogeneous across studies, including DSM-based diagnoses in administrative datasets, clinical psychiatric evaluation, and, in some cases, validated rating scales; however, several studies did not specify the assessment tool used, limiting direct comparability. Moreover, biological data supporting mechanistic hypotheses are still emerging, with several studies based on small clinical samples or preclinical models. Despite these limitations, the convergence of epidemiological observations, clinical reports, and biological findings provides a coherent framework that justifies further investigation into depression as a potential early manifestation of PDAC.

## 5. Conclusions

Depression is a frequent and clinically significant condition in patients with PDAC, with implications that extend beyond a mere psychological reaction to cancer diagnosis. Available evidence suggests that depressive symptoms may precede the clinical diagnosis of PDAC, emerging as a potential prodromal manifestation sustained by complex biological mechanisms such as systemic inflammation, neuroendocrine alterations, and diversion of the tryptophan–kynurenine pathway.

From a clinical perspective, depression is consistently associated with poorer quality of life, lower treatment adherence, and reduced survival, exerting a substantial influence on the disease trajectory. These findings underscore the need to systematically incorporate psychiatric assessment into PDAC care pathways.

Future directions should focus on investigating and validating depression as an early marker through large prospective cohorts, integrating clinical, biological, and digital indicators. At the same time, the exploration of innovative therapeutic strategies—such as immuno-metabolic interventions and IDO1 inhibition—may open new avenues for addressing oncological and psychiatric outcomes in a targeted manner. Ultimately, a multidisciplinary approach integrating oncology, psychiatry, and psycho-oncology appears essential to improving prognosis and quality of life in this particularly vulnerable population.

## Figures and Tables

**Table 1:** Epidemiological and clinical studies on prodromal depression in PDAC.

Author (Year)	Study Design/Data Source	N of Patients	Timing of Depression	Main Findings
[8] Shakin & Holland (1988)	Clinical review/case series	–	Preceding and concomitant	High prevalence of depression in PC patients compared to other cancers.
[7] Makrilia et al. (2009)	Narrative review	–	Preceding and concomitant	Evidence of depression as a possible biologically mediated paraneoplastic syndrome.
[3] Olson et al. (2016)	Case–control study (Memorial Sloan Kettering)	~500	Prior to diagnosis	Fatigue and depression are significantly more common in PC patients, independent of weight loss and diabetes.
[11] Seoud et al. (2020)	National population-based study	>3000	Before and after diagnosis	16.4% with pre-diagnosis depression vs. 13% post-diagnosis; depression may precede PC.
[10] Ferreira et al. (2021)	Case reports/clinical analyses	3 cases	Prior to physical symptoms	Depressive episodes as the first manifestation of PC, leading to diagnostic delay.
[12] Davis et al. (2022)	Multicenter prospective study	250	Prodromal (within 6 months)	Depression and anxiety associated with lower treatment adherence and reduced survival.
[13] Ji et al. (2023)	Longitudinal study	217	Within 6 months of diagnosis	Depressive disorders → 1-year mortality: 30% in depressed vs. 10% in non-depressed patients.
[4] Boyd et al. (2012)	SEER–Medicare study (U.S. cohort)	23,745	Preexisting	Depression is associated with more advanced stage, lower likelihood of surgery/chemotherapy, and reduced overall survival.

Key epidemiological and clinical studies documenting the association between depression and pancreatic cancer, with particular attention to the prodromal phase.

## Data Availability

No new data were created or analyzed in this study. Data sharing is not applicable to this article.

## Abbreviations

The following abbreviations are used in this manuscript:

PDAC	Pancreatic ductal adenocarcinoma
KYN	kynurenine
PC	Pancreatic Cancer
PNET	pancreatic neuroendocrine tumor
